# Functional Analysis of Genes Involved in Glycerolipids Biosynthesis (*GPAT1* and *GPAT2*) in Pigs

**DOI:** 10.3390/ani9060308

**Published:** 2019-05-31

**Authors:** Ilona Mitka, Katarzyna Ropka-Molik, Mirosław Tyra

**Affiliations:** 1 Department of Pig Breeding, National Research Institute of Animal Production, Poland, 1 Krakowska, 32-083 Balice, Poland; miroslaw.tyra@izoo.krakow.pl; 2 Department of Animal Molecular Biology, National Research Institute of Animal Production, Poland, 1 Krakowska, 32-083 Balice, Poland

**Keywords:** triacylglycerol synthesis, *GPAT1*, *GPAT2*, intramuscular fat, pig

## Abstract

**Simple Summary:**

Pork consumption is the highest among all meats in Poland and in the world. Current breeding programs were designed to obtain high meat content and low levels of fat in pork carcasses. This resulted in a decrease in the quality of meat. Numerous researchers indicated that intramuscular fat content (IMF) is the determining factor for meat quality and consumer’s acceptance of meat. The genes *GPAT1* and *GPAT2*, being the objective of this study are involved in triacylglycerol (TAG) synthesis. TAGs are the main constituents of animal fat as well as of IMF. The aim of this study was to assess the expression level of the *GPAT1* and *GPAT2* genes in musculus longissimus lumborum, subcutaneous fat and liver of pigs. Moreover, association analysis between the genes’ expression, production traits, quality and sensory parameters of pork was carried out. The results obtained showed significant differences in the mRNA expression of analyzed genes between tissues and breeds of pigs. Furthermore, association analysis showed significant associations between expression level of the genes and some of the production traits, sensory and quality parameters of pork. The results of this study indicated the possibility of modification of desired traits through transcriptional control of gene expression.

**Abstract:**

Glycerol-3-phosphate acyltransferase (GPAT) enzymes catalyze the first step in triacylglycerol (TAG) synthesis. Genes that belong to the *GPAT* family are potential genetic markers for intramuscular fat content (IMF) content and thus meat quality. The objective of this study was to analyze the expression of *GPAT1* and *GPAT2* genes in musculus longissimus lumborum, liver and subcutaneous fat of various breeds of pigs. Furthermore, correlations between the genes’ expression abundance and utility traits, meat quality and meat texture parameters of pork were determined. The results obtained showed significant differences in the mRNA level of *GPAT1* between analyzed tissues and breeds. The highest expression of *GPAT1* gene was observed in liver tissue (*p* ≤ 0.01). Furthermore, significantly higher *GPAT1* transcript level in the m. longissimus lumborum was observed for duroc in comparison to other analyzed breeds (*p* ≤ 0.05). Expression of the *GPAT2* gene was shown only in the liver tissues, however statistically significant differences between the analyzed breeds were not observed. Correlation analysis confirmed the highest association between *GPAT2* gene expression level in liver and cohesiveness and resilience traits of m. longissimus lumborum (*p* ≤ 0.01).

## 1. Introduction

The glycerol-3-phosphate acyltransferase 1 (*GPAT1*) and glycerol-3-phosphate acyltransferase 2 (*GPAT2*) genes belong to the *GPAT* gene family. The genes encode glycerol-3-phosphate acyltransferase enzymes, which play an essential role in the biosynthesis of triacylglycerol (TAG) which is the primary unit of energy storage in mammalian cells. The *GPAT* enzymes initiate the pathway of TAG synthesis by catalyzing the esterification of long-chain acyl-CoAs at the sn-1 position of glycerol-3-phosphate and resulting in the production of lysophosphatidic acid (LPA) [1].

*GPAT1* gene (also known as *GPAM*) is the most widely studied and described in the *GPAT* gene family. *GPAT1* contributes up to 50% of the total *GPAT* activity in the liver and it is regulated depending on the nutritional status of the animal [2]. Studies on knockout mice have demonstrated reduced body weight, liver and plasma TAG content, as well as reduced secretion of low-density lipoprotein (VLDL) of GPAT1-/- females [3]. *GPAT1* is highly expressed in adipose tissue and liver which are the major sites of TAG synthesis [4]. Based on the function and previous studies, the *GPAT1* gene is considered to be a genetic marker for prediction of intramuscular fat (IMF) content in musculus longissimus dorsi (MLD) [5]. Primary pork cuts (like loin) are leaner than other cuts, so the problem of low IMF content applies mostly to MLD.

The research performed to date demonstrate that the expression of *GPAT2* was detected in many tissues, but its level was at least 50-fold higher in testis [4]. Furthermore, in contrast to *GPAT1*, mRNA expression of *GPAT2* does not change as a consequence of fasting or refeeding [4].

In the absence of literature concerning *GPAT1* and *GPAT2* gene function in lipogenesis and fat deposition in pigs, the aim of this study was to assess the expression of *GPAT1* and *GPAT2* genes in m. longissimus lumborum, liver and subcutaneous fat of various breeds of pigs raised in Poland. In addition, correlations between the genes’ expression abundance and production traits as well as sensory parameters of pork were determined.

## 2. Materials and Methods

### 2.1. Animals

The present study involved 47 sows belonging to five breeds of pigs raised in Poland: polish large white—PLW (*n* = 8), polish landrace—PL (*n* = 3), pulawska (*n* = 16), pietrain (*n* = 8), duroc (*n* = 12). All animals were kept at the Pig Performance Testing Stations in Pawlowice and Chorzelow under the same housing and feeding conditions. The performance test started when the pigs reached 30 kg and finished when they had reached 100 kg (±3) live weight. All pigs were slaughtered at the same live weight in order to obtain the most reliable and comparable results for carcass traits and meat characteristics. The age of pigs was from 170 (for duroc) to 188 (for pulawska) days—average 175 days (due to breed specific differences in growth and fattening rates). The differences between breeds (in age at point of slaughter) were not statistically significant. The sampling of biological material does not require the approval of Animal Experimentation committee, because samples were collected in slaughterhouses from pigs which were slaughtered, dissected and after carcass evaluation, meat was standard intended for consumption.

### 2.2. Tissue Samples and Carcass Characteristics

Tissue samples of m. longissimus lumborum, subcutaneous fat and liver were collected in tubes containing RNAlater™ Stabilization Solution (ThermoFisher Scientific, Waltham, MA, USA) up to 20 min after slaughter and stored at −20 °C.

All carcass characteristics were evaluated on the basis of the Pig Performance Testing Station procedure [6]. Food intake was recorded throughout the test. After slaughter and a 24 h chill, the right side of each carcass was dissected. The slaughter performance (%) was calculated as a ratio of carcass mass to the pre-slaughter body weight of the animal. The individual carcass cuts (including loin and ham) were weighed as well as loin width, height and area were measured. Carcass length was measured from the first rib at the breast bone to the front edge of the pubic symphysis. Backfat thickness was shown as an average from five measurement (including measurements: at the thickest point over the shoulder, on the back above the joint between the last dorsal vertebra and the first lumbar vertebra, at three points on the back—rostral (vertebra 1), middle (vertebra 2), caudal (vertebra 3) of the gluteus muscle section). Carcass meat content was calculated using the formula: y = 1.745x_1_ + 0.836x_2_ + 0.157x_3_ − 1.884, where: y is the calculated meat content (kg), x_1_ is the ham without skin and backfat (kg), x_2_ is the loin without backfat + tenderloin (kg), x_3_ is the double width + height of loin eye (2A + B) (cm). 

The intramuscular fat (IMF) content in m. longissimus lumborum samples was determined as crude fat by Soxhlet extraction with fat solvents (Soxtherm SOX 406, Gerhardt). Water holding capacity (WHC) was measured by the compression method [7]. Meat color was evaluated with the use of CR-310 Chroma Meter (Konica Minolta, Tokyo, Japan). The pH was measured in musculus longissimus lumborum and musculus semimembranosus, 45 min (pH45) and 24 h (pH24) post-mortem using Matthäus, pH-Star CPU (Matthäus, Pöttmes, Germany).

The meat texture parameters were analyzed on homogenous m. longissimus lumborum samples using TA.XTplus Texture Analyser (Stable Micro Systems Ltd., Goldaming, UK). The muscle slices for this analysis were 3.5 cm wide and weighed approximately 200 g. Warner–Bratzler shear force (WBS) was measured for both raw and cooked meat. Texture profile analysis (TPA) parameters such as hardness, springiness, cohesiveness, chewiness, and resilience were evaluated on cooked meat samples with the use of the Miniature Kramer Shear/Ottawa Cell attachment. 

### 2.3. RNA Isolation and Reverse Transcription

Total RNA was isolated from tissue samples using three different methods, because of the high specificity of the tissue towards the isolation method. Total RNA from m. longissimus lumborum was extracted with the use of TRI-Reagent (Sigma-Aldrich, Poznan, Poland), according to the method described by Chomczynski [8]. Total RNA from subcutaneous fat was isolated using Syngen Tissue RNA Mini Kit (Syngen, Wroclaw, Poland), whereas from the liver with the use of PureLink^®^ RNA Mini Kit (ThermoFisher Scientific, Waltham, MA USA), following the manufacturer’s instructions. The concentration and quality of total RNA were checked by UV spectrometry (NanoDropTM2000, ThermoFisher Scientific, Waltham, MA USA) and by agarose electrophoresis, respectively. Moreover, RNA integrity was evaluated using TapeStation 2200 (Agilent) and RNA ScreenTape (Agilent). The RNA integrity number (RIN) values were as follows: an average 8.3 for fat tissue, 6.9 for liver and 7.5 for muscle tissue.

Total RNA (0.15 μg/150 ng) was reverse-transcribed into cDNA using a TranscriptMe RNA Kit (DNA-Gdansk, Poland), according to the manufacturer’s instructions.

### 2.4. Real-Time PCR Analysis

The amount of mRNA for *GPAT1* and *GPAT2* genes was quantified on a Quant Studio 7 Flex Real-Time PCR System with the use of a TaqMan Gene Expression Master Mix (Applied Biosystems, Warsaw, Poland). The real-time quantitative PCR analyses were performed in three repeats for each sample and in two reaction mixes: *GPAT1*/*RPLS27* and *GPAT2*/*RPS29*, in a total reaction volume of 21 μL. The first reaction mix contained: 12.5 μL TaqMan Gene Expression Master Mix, 1 μL of *GPAT1* assay, 0.33 μL of *RPL27* assay, 3.1 μL of PCR water, 1 μL of cDNA. The second reaction mix contained: 12.5 μL TaqMan Gene Expression Master Mix, 0.5 μL of *GPAT2* probe, 0.5 μL of *GPAT2* primers, 0.33 μL of *RPS29* assay, 6.17 μL of PCR water and 1 μL of cDNA. The thermal cycling conditions were as follows: 50 °C for 2 min (Uracil-DNA Glycosylase (UDG) incubation), 95 °C for 10 min (AmpliTaq, UP enzyme activation) and 50 cycles of PCR: 95 °C for 15 s (denaturation) and 60 °C for 1 min (annealing/extension). The results were analyzed with the use of QuantStudio Real-Time PCR Software v1.2 (Applied Biosystems, California, USA).

Primers and probes were designed using Primer Express 3.0 software (Applied Biosystems, California, USA) (Table 1). For each gene, the efficiency of real-time PCR reactions was estimated using a standard curve method. The comparative delta-delta Ct (∆∆Ct) method was used to determine relative fold changes in gene expression. All data were normalized with the housekeeping genes: *RPL27* and *RPS29* selected based on previous reports [9,10] and the geNorm optimization method.

### 2.5. Statistical Analyses

Statistical analysis was performed using the general linear model (GLM) procedure of SAS (SAS Institute, Cary, NC, v.8.02,2001). The linear model had the following form:

Y*_ijk_* = μ + TS*_i_* + BT*_j_* + e*_ijk_*

where:

Y*_ijkl_*—trait value,

μ—general mean,

TS*_i_*—constant effect of the Pig Performance Testing Station,

BT***_j_***—constant effect of analyzed breed or tissue,

e*_ijk_*—random error.

The significance of differences between groups within the analyzed effect was estimated using the Tukey–Kramer test.

Correlation between gene expression abundance and selected utility traits (slaughter and fattening) as well as with meat quality and meat texture parameters, were calculated using the CORR procedure (Pearson correlation coefficient) (SAS Institute, Cary, NC, USA, v.8.02, 2001).

## 3. Results

### 3.1. Expression Levels of *GPAT1* and *GPAT2* Genes in Analyzed Tissues

**Figure 1:**
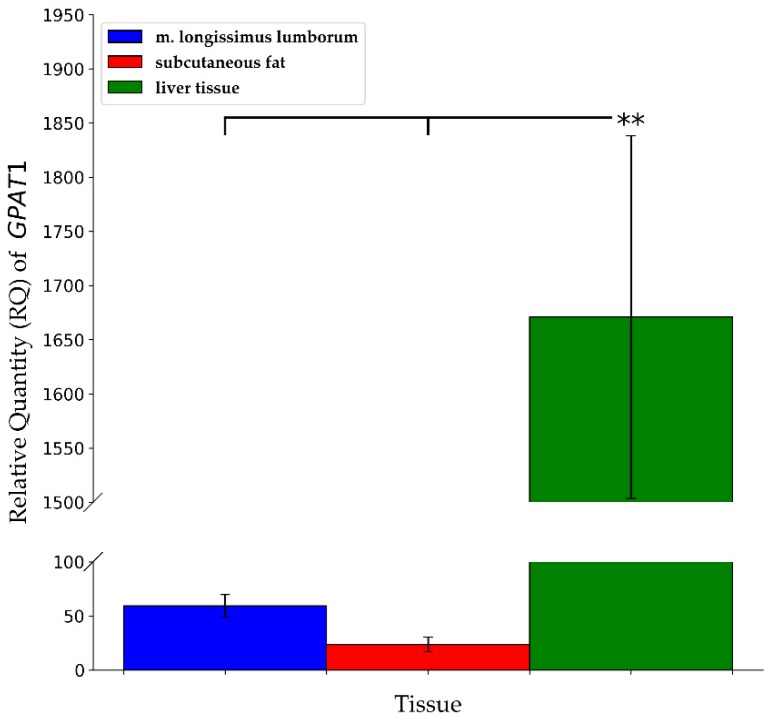
Relative quantity (RQ) of *GPAT1* transcript abundance in different tissues (m. longissimus lumborum, subcutaneous fat, liver tissue) across all breeds together (** *p* ≤ 0.01). Data are presented as means ± SEM (standard error of the mean).

The mRNA expression of *GPAT1* was significantly different between analyzed tissues (Figure 1). The highest *GPAT1* gene expression level was observed in the liver tissue (*p* ≤ 0.01). Furthermore, it was more than 70-fold higher than in subcutaneous fat and more than 28-fold higher than in musculus longissimus lumborum. The expression of the *GPAT2* gene was shown only in the liver tissue.

### 3.2. The mRNA Expression of *GPAT1* between Tissues within Studied Breeds

**Figure 2:**
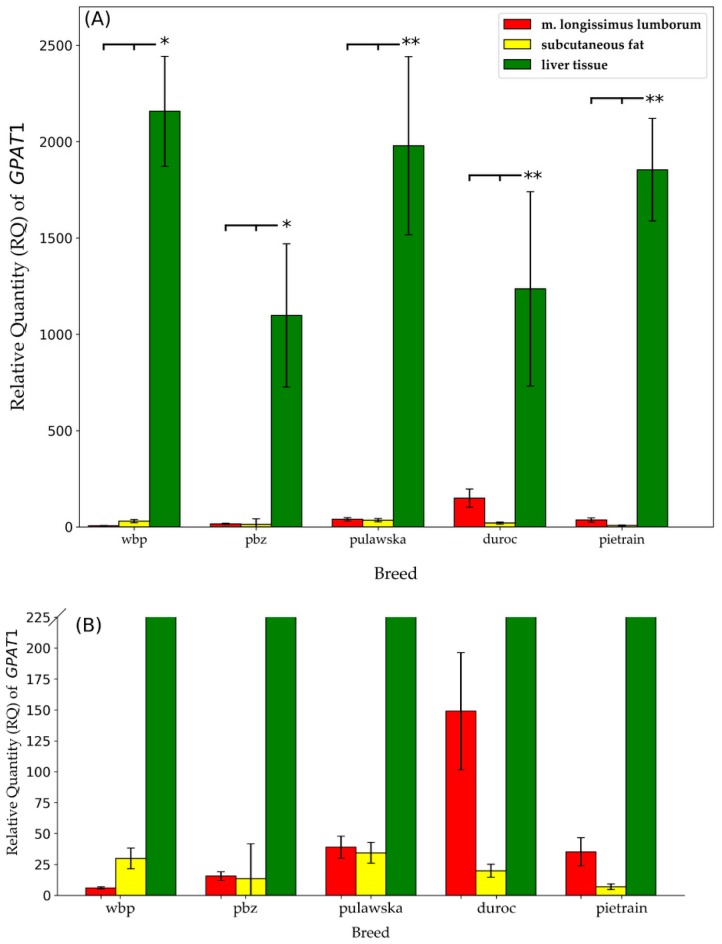
Relative quantity (RQ) of *GPAT1* transcript abundance in m. longissimus lumborum, subcutaneous fat, liver tissue across all pigs within each breed (* *p* ≤ 0.05; ** *p* ≤ 0.01). Data are presented as means ±SEM. (**A**) Full chart; (**B**) zoomed in chart with y-axis ranging from 0 to 225.

As shown in Figure 2A,B, significant differences were observed in the mRNA expression of *GPAT1* between tissues within analyzed breeds. The highest *GPAT1* gene expression level was observed in the liver tissue of all studied breeds. For breeds representing the maternal component (PLW and PL) the significance level of *p* ≤ 0.05 was demonstrated. On the other hand, for breeds representing the paternal component (duroc and pietrain) as well as for pulawska breed which constitutes a reserve of genetic diversity, the statistical significance was shown on the level of *p* ≤ 0.01. Furthermore, the lowest expression of *GPAT1* was observed in subcutaneous fat for all analyzed breeds apart from PLW. For PLW the lowest level of *GPAT1* mRNA expression was observed in m. longissimus lumborum.

### 3.3. *GPAT1* and *GPAT2* Gene Expression between Breeds within Analyzed Tissues

**Figure 3:**
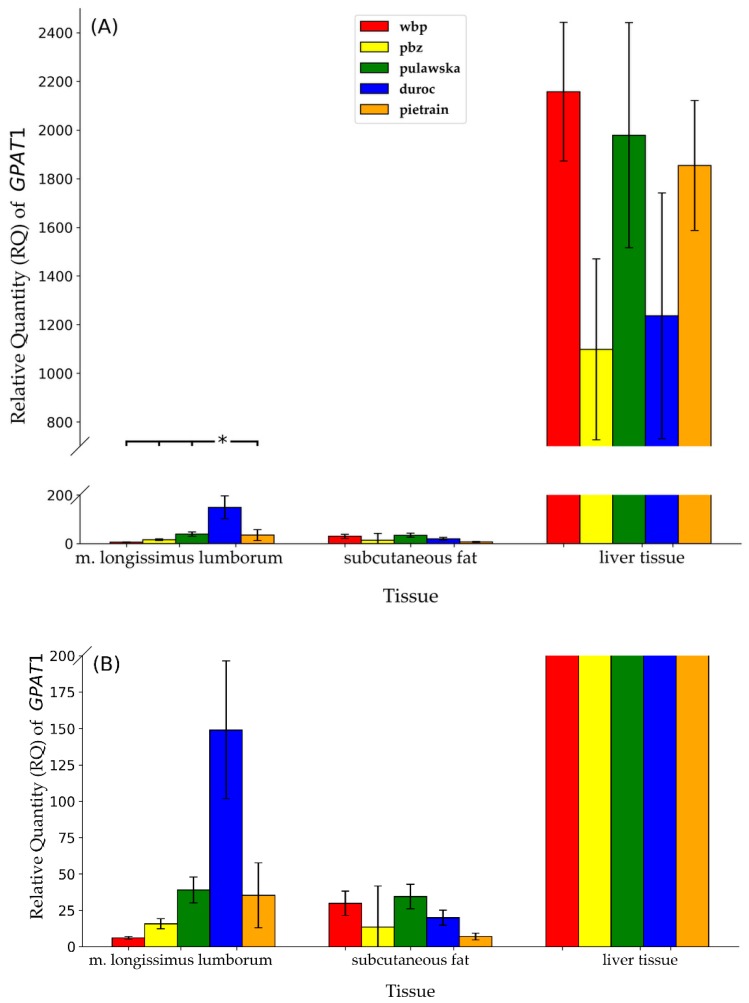
Relative quantity (RQ) of *GPAT1* transcript abundance between all individuals within breeds in analyzed tissues: m. longissimus lumborum subcutaneous fat, liver tissue (** *p* ≤ 0.01). Data are presented as means ±SEM. (**A**) Full chart; (**B**) zoomed in chart with y-axis ranging from 0 to 200.

**Figure 4:**
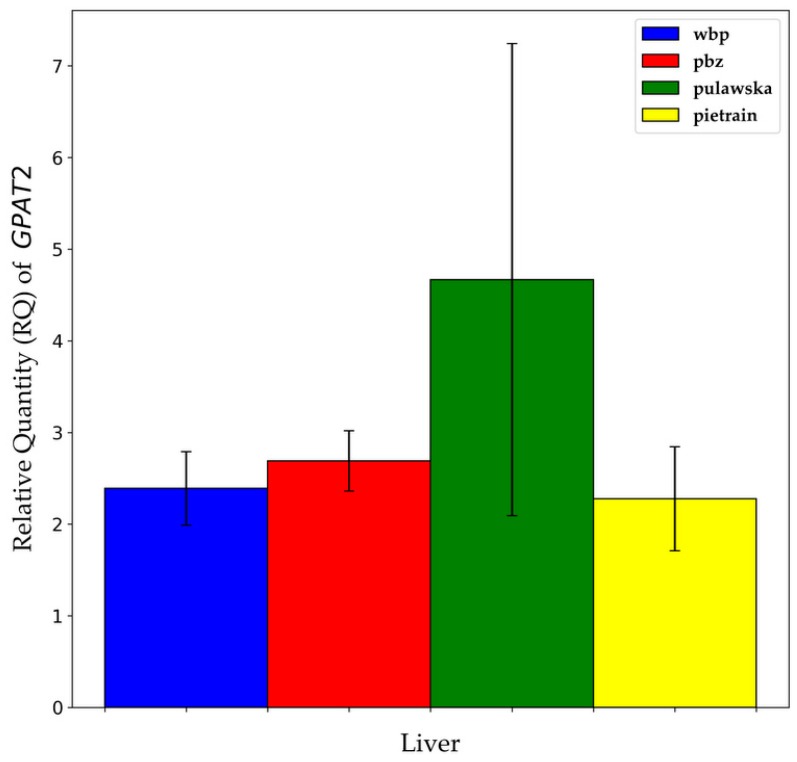
Relative quantity (RQ) of *GPAT2* transcript abundance between different breeds of pigs within liver tissue. Data are presented as means ±SEM.

Statistically significant variations in the mRNA expression of *GPAT1* between breeds were established only in m. longissimus lumborum (Figure 3A,B). The highest gene expression was noted in the m. longissimus lumborum of duroc in comparison to the rest analyzed breeds of pigs (*p* ≤ 0.05). The mRNA abundance of *GPAT1* in subcutaneous fat was the highest for pulawska, while in liver tissue for PLW however, statistical analysis did not show any significant differences in the transcript level, probably due to the high variability between individuals within a group. As mentioned above, the *GPAT2* gene expression was observed only in liver tissue but not in m. longissimus lumborum as well as not in subcutaneous fat. As shown in Figure 4, the amount of transcript for the *GPAT2* gene in liver tissue was the highest for pulawska and the lowest for pietrain pigs. Nevertheless, no significant difference was observed in the mRNA expression of *GPAT2* between analyzed breeds of pigs.

### 3.4. Correlation between the Expression Abundance of *GPAT1* and *GPAT2* Genes and Utility Traits in Pigs

Correlation analyses between the genes’ expression and slaughter traits, fattening traits as well as meat quality and meat texture parameters were calculated with the use of a Pearson correlation. The amount of *GPAT1* gene in m. longissimus lumborum was significantly correlated with loin weight, loin eye height and WB shear force of cooked meat (r = −0.277, r = −0.212, r = −0.270, respectively, *p* ≤ 0.05) (Table 2 and Table 3). Expression level of the *GPAT1* gene in subcutaneous fat was highly negatively correlated (*p* ≤ 0.05) with meat color a* (redness) (r = −0.305), following TPA parameters: springiness (r = −0.408), cohesiveness (r = −0.358), resilience (r = −0.334) (Table 3 and Table 4), and highly positively correlated with loin eye width (r = 0.257) and the age at slaughter (r = 0.246, *p* ≤ 0.05). The weight of loin without skin and backfat, meat content in carcass and meat mass in particular primary cuts (r = −0.265 r = −0.228 r = −0.261, respectively, *p* ≤ 0.05) showed negative correlations with expression abundance of the *GPAT1* gene in liver tissue. While IMF content and following TPA parameters: springiness, cohesiveness, resilience showed positive correlations (r = 0.285, r = 0.292, r = 0.229, r = 0.229, respectively, *p* ≤ 0.05). The highest number and the strongest correlations were observed between the expression level of the *GPAT2* gene in liver tissue and some of the analyzed traits. For meat color (a*) (r = 0.306), longissimus dorsi muscle pH 45 min after the slaughter (r = 0.323), springiness (r = 0.503), daily live gain (r = −0.302), age at slaughter (r = 0.363), the number of days of control fattening from 30 kg to 100 kg (r = 0.424) significant correlations were shown (*p* ≤ 0.05) (Table 3, Table 4 and Table 5). While for cohesiveness and resilience (r = 0.772, r = 0.833, respectively, *p* ≤ 0.01) strong positive correlations were observed. No significant relationship was observed between the amount of transcript of these genes and the rest of the analyzed parameters.

## 4. Discussion

*GPAT1* and *GPAT2* catalyze the first step of the triglyceride biosynthesis and thus play a crucial role in the pathway [11,12]. In the research carried out on pigs, it was proved that an increase in IMF content is mainly the cause of an increase of TAG content [13]. The first research on the *GPAT1* and *GPAT2* genes was focused on finding their function. In recent years more research has been conducted to analyze the structure, search for polymorphisms in these genes and to estimate mRNA abundance. However, the number of studies carried out to determine expression levels of *GPAT1* and *GPAT2* genes is still limited. Thus in our study, we analyzed the expression level of *GPAT1* and *GPAT2* genes in a few tissues of various breeds of pigs in Poland demonstrating a significant difference in relation to meatiness, fatness and other production traits.

The analysis of *GPAT1* gene expression demonstrated significantly higher transcript abundance in m. longissimus lumborum for duroc pigs (*p* ≤ 0.05). It should be pointed out that duroc breed is characterized by the highest amount of IMF in m. longissimus lumborum among all the breeds of pigs raised in Poland [14]. Furthermore, even though no statistical differences were shown between large white and landrace breeds, the mRNA abundance of *GPAT1* in subcutaneous fat and liver for PL was about 50% lower than for PLW. The reverse situation between these breeds was established in m. longissimus lumborum, where a two-fold higher level of *GPAT1* mRNA was shown for polish landrace compared to the polish large white. PLW and PL breeds constitute the maternal component. They are used in commercial crossing and they are also characterized by similar values for utility, slaughter as well as for meat quality traits. The lowest transcription level of the *GPAT1* gene in subcutaneous fat was shown for pietrain (which is characterized by the lowest fat content) while the highest for pulawska (a breed of pigs characterized with the highest fat content and the lowest meatiness). Pigs of PLW, pulawska, pietrain breeds demonstrated a similar amount of *GPAT1* mRNA in liver and it can be clearly seen that it is higher than for the rest of analyzed breeds of pigs (PL, duroc). Furthermore, the expression level of the *GPAT1* gene significantly depends on the type of tissue. The highest *GPAT1* expression abundance was observed in the liver (1670.8) and it was highly significantly higher in comparison to m. longissimus lumborum (59.3) and subcutaneous fat (23.5) (*p* ≤ 0.01).

The analysis has demonstrated the highest value of mRNA for *GPAT1* gene in the liver. In the pig atlas dataset (where *GPAT1* is included as pseudonym *GPAM*) the highest expression in the liver relative to other tissues was shown, which reflects the findings from this study [15,16]. Similar results were also observed in the studies carried out by Bertolesi et al. [17] on the model organism *Xenopus laevis*, where *GPAT1* gene expression was observed mainly in the liver. Furthermore, the highest expression of *GPAT1* was also shown in mice [18] and rat [19] and it was higher than in muscle and fat tissue. However, there is also research in which different results and trends were established. 

In contrast to our results, Wang et al. [4] demonstrated the highest mRNA expression of the *GPAT1* in white and brown adipose tissue. Furthermore, it was around two-fold higher than in liver and three-fold higher than in soleus muscle. Similarly, in humans *GPAT1* mRNA was most highly expressed in adipose tissue and it was even more than 10-fold higher than in other tissues [20]. From among all farm animals, the expression of *GPAT1* gene was also determined for cattle and chicken. The highest expression level of *GPAT1* in Angus cattle was obtained in adipose tissue and it was over 20-fold higher than in skeletal muscle and in the liver [21]. Moreover, the offspring of bulls with low residual feed intake (RFI) also showed reduced *GPAM* gene expression. These results confirmed the down-regulation of a regulatory network controlling fat deposition and thus suggested reduced body fat content among those animals. In the microarray analysis carried out on chicken, Claire D’Andre et al. [22] have found the highest levels of *GPAM* mRNA expression in abdominal and subcutaneous fat, while it was only rarely detectable in the liver. Additionally, the comparison analysis of *GPAM* gene expression level between fast-growing White Recessive Rock (WRR) chickens and slow-growing Xinghua chickens was conducted in this study. The results obtained by Claire D’Andre and his co-workers showed that the expression of *GPAM* was down-regulated in abdominal fat and pituitary tissue, whereas it was up-regulated in abdominal fat and breast muscle tissue of fast-growing (WRR) males. The results of this research suggest that the differences in the expression level of the lipid metabolism genes (including *GPAM*) might be one of the factors responsible for fat deposition between fast- and slow-growing chickens at the developmental stage [22].

There are also other studies that indicated the association of *GPAT1* gene expression with utility traits in farm animals. Analyses carried out by Bionaz and Loor [23] on Holstein dairy cows showed a high mRNA abundance of *GPAT1* in the mammary gland during lactation. Moreover, it was at least two-fold higher than for other genes encoding enzymes, which catalyze further steps of the TAG biosynthesis pathway. Similarly, expression of the *GPAM* gene was significantly up-regulated in the mammary tissue of lactating yaks [24]. The results of these studies confirm the increased activity of the *GPAM* enzyme and it’s an important role in TAG biosynthesis. The crucial role of *GPAM* gene in the regulation of cellular TAG and phospholipids levels was shown in in vitro studies carried out by Yu et al. [25] on bovine embryonic fibroblast (BEF) cells with *GPAM* gene silencing and over-expression. In BEF cells after silencing of *GPAM* gene, a significant decrease in TAG synthesis, as well as down-regulated expression of genes related to lipid metabolism pathway (*AGPAT1*, *AGPAT3*, *AGPAT4*, *AGPAT6*) was observed (*p* ≤ 0.05). The opposite situation i.e., increased TAG biosynthesis and up-regulated expression of lipid metabolism-related genes was observed in BEF cells after over-expression of *GPAM* gene (*p* ≤ 0.05) [25]. These results suggest an essential role of *GPAM* for TAG synthesis. Furthermore, *GPAM* might also regulate the mRNA expression of other genes related to lipid metabolism (e.g., *AGPATs*, which are key enzymes involved in the second step of TAG synthesis).

In this study, the mRNA expression of *GPAT2* gene was shown only in the liver, while in m. longissimus lumborum and backfat it was not found. Although no statistical differences in the *GPAT2* gene expression level between analyzed breeds of pigs were determined, the highest mRNA expression in the liver was observed for pulawska (a breed that constitutes a genetic reserve). It should be emphasized that the mean expression level of *GPAT2* in the liver amounted only to 3.35. Similar results were observed in the studies carried out by Bertolesi et al. [17] on *Xenopus laevis*, where *GPAT2* gene expression was on the very low or even almost undetectable level in the liver, whereas high *GPAT2* gene expression level was shown in gonadal tissue during early organogenesis. These data suggest a possible role of *GPAT2* gene as a sex-determining genetic marker, specific for testis [17]. *GPAT2* gene expression analysis carried out on mice also showed very high mRNA abundance in testis and it was even 50-fold higher than in other tissues (e.g., liver and brown adipose tissue) [4]. However, in the pig atlas dataset expression of *GPAT2* was shown across many tissues in pigs [15,16].

An important part of the present study was correlation analysis between *GPAT1* and *GPAT2* genes expression and slaughter traits, fattening traits as well as meat quality and meat texture parameters. The mRNA abundance of the *GPAT2* gene in the liver showed the greatest correlation with the following TPA parameters of m. longissimus lumborum: cohesiveness (r = 0.772) and resilience (r = 0.833) (*p* ≤ 0.01). Significant correlations of mRNA expression of the *GPAT1* gene with some of the analyzed traits were also observed. However, no significant correlations were showed between mRNA abundance of *GPAT1* in m. longissimus lumborum and meat quality traits. In the study carried out by Jeong et al. [5] on Korean cattle steers, a strong positive correlation between mRNA expression of the *GPAT1* in m. longissimus dorsi and IMF content was demonstrated (r = 0.74; *p* ≤ 0.001). Furthermore, multiple regression analysis showed that the mRNA abundance of the *GPAT1* gene in m. longissimus dorsi was the major factor in predicting IMF content (54%) among all of the analyzed lipid metabolic genes (*ACC*, *FASN*, *LPL*, *CD36*, *FATP1*, *GPAT1*, *AGPAT1*, *DGAT1*, *DGAT2*, *ATGL*, *HSL*, *MGL*, *CPT1B*, *VLCAD*, *MCAD*). This study has a substantial impact on determining the IMF content in m. longissimus dorsi of Korean cattle and thus suggesting that *GPAT1* gene should be considered as a genetic marker to predict IMF deposition.

## 5. Conclusions

This is the first study focusing on the mRNA abundance of the *GPAT1* and *GPAT2* genes in pigs. The gene expression measurements showed high individual variation of the transcript level of both genes, especially in liver tissue. The mRNA abundance of *GPAT1* and *GPAT2* genes depends mainly on the tissue type rather than the analyzed breed. The expression of the *GPAT1* gene in the liver was high and had a positive influence on the IMF level in the loin. However, at the same time, it had a negative impact on the meatiness traits (loin weight, carcass meat content, meat mass in particular primary cuts) as well as sensory parameters of meat (springiness, cohesiveness, resilience). A similar trend was observed for *GPAT2* gene but with less impact. Increasing expression of *GPAT1* gene in backfat resulted in advantage changes in terms of meat quality, meat texture and slaughters parameters. The simultaneously untoward effect was observed with regard to fattening traits.

The results regarding the association analysis of the *GPAT1* and *GPAT2* genes expression with utility traits seem to be promising. They indicate the possibility of modification of desired traits through transcriptional control of gene expression. However, genetic mechanisms underlying fat synthesis and deposition are not fully understood. Therefore, further research on this topic is still required.

## Figures and Tables

**Table 1:** Primers and probes used in real-time PCR.

Gene (Accession no.)	Primer Sequence	Probe Sequence	Amplicon Size, bp
*GPAT1 (NC_010456.4)*	TaqMan Gene Expression Assay ID: Ss03373682_m1 (Applied Biosystems)	(FAM)	*104*
*GPAT2 (NC_010445.3)*	F: GGAAATACCGCCCTTTTGTG R: GCAGAAGCCTAGATCCATTATGC	CACCCCTAAGAGCTGG (NED)	*98*
*RPL27 (NM_001097479)*	F: CGCTACTCCGGACGCAAA R: GGTCTGAGGTGCCATCATCA	CGGTCATCGTAAAGAA (VIC)	*58*
*RPS29 (NM_001001633)*	F: CGGAAATACGGCCTCAATATG R: GCCAATATCCTTCGCGTACTG	CCGCCAGTGCTTC (VIC)	*60*

**Table 2:** Correlation between the expression level of the *GPAT1* gene in m. longissimus lumborum, subcutaneous fat, liver tissue and the expression level of *GPAT2* gene in liver tissue with slaughter traits (* *p* ≤ 0.05; ** *p* ≤ 0.01).

Gene/Tissue	*GPAT1*			*GPAT2*
Slaughter Traits	m. longissimus lumborum	Subcutaneous Fat	Liver Tissue	Liver Tissue
Slaughter performance (%)	−0.185	0.083	−0.088	0.174
Loin weight (kg)	−0.277 *	0.023	−0.115	0.187
Loin weight without skin and backfat (kg)	−0.192	0.034	−0.265 *	0.033
Ham weight without skin and backfat (kg)	−0.146	−0.081	−0.234	−0.094
Carcass length (cm)	−0.058	0.042	0.059	0.045
Mean backfat thickness from five measurements (cm)	0.087	0.024	0.150	0.068
Loin eye width (cm)	0.030	0.257 *	−0.056	−0.112
Loin eye height (cm)	−0.212 *	−0.080	−0.074	−0.034
Loin eye area (cm^2^)	−0.140	0.004	−0.220	−0.076
Lean meat content (%)	−0.022	−0.072	−0.228 *	−0.219
Weight of meat from primal cuts (kg)	−0.177	−0.015	−0.261 *	−0.082

**Table 3:** Correlation between the expression level of the *GPAT1* gene in m. longissimus lumborum, subcutaneous fat, liver tissue and the expression level of *GPAT2* gene in liver tissue with meat texture parameters (* *p* ≤ 0.05; ** *p* ≤ 0.01).

Gene/Tissue	*GPAT1*			*GPAT2*
Meat Texture Parameters	m. longissimus lumborum	Subcutaneous Fat	Liver Tissue	Liver Tissue
WB_R_FIR	0.032	−0.048	0.049	0.003
WB_R_TUG	-0.081	−0.047	0.050	−0.022
WB_C_FIR	−0.270 *	−0.089	−0.038	−0.002
WB_R_TUG	−0.091	−0.115	0.111	−0.126
TPA_ hardness	0.012	−0.059	0.035	−0.115
TPA_ springiness	0.049	−0.408 *	0.292 *	0.503 *
TPA_ cohesiveness	0.033	−0.358 *	0.229 *	0.772 **
TPA_ chewiness	0.035	−0.156	0.116	−0.006
TPA_ resilience	0.080	−0.334 *	0.229 *	0.833 **

WB: Warner–Bratzler test: (R—raw; C—cooked meat); (FIR—firmness; TUG—toughness); TPA: texture profile analysis.

**Table 4:** Correlation between the expression level of the *GPAT1* gene in m. longissimus lumborum, subcutaneous fat, liver tissue and the expression level of *GPAT2* gene in liver tissue with meat quality parameters (* *p* ≤ 0.05; ** *p* ≤ 0.01).

Gene/Tissue	*GPAT1*			*GPAT2*
Meat Quality Parameters	m. longissimus lumborum	Subcutaneous Fat	Liver Tissue	Liver Tissue
Intramuscular fat content (IMF; %)	0.053	0.066	0.285 *	0.165
Water holding capacity (%)	0.196	0.112	−0.207	−0.030
Meat color (L *)	−0.080	0.074	0.163	0.260
Meat color (a *)	0.109	−0.305 *	0.132	0.306 *
Meat color (b *)	0.073	−0.120	0.183	0.246
45 min post-mortem pH in MLD	−0.151	0.083	0.148	0.323 *
24 h post-mortem pH in MLD	0.028	0.043	−0.187	0.072
45 min post-mortem pH in SEMI	−0.228	−0.202	0.205	0.047
24 h post-mortem pH in SEMI	−0.058	0.090	0.025	−0.289

MLD—m. longissimus dorsi; SEMI—m. semimembranosus.

**Table 5:** Correlation between the expression level of the *GPAT1* gene in m. longissimus lumborum, subcutaneous fat, liver tissue and the expression level of *GPAT2* gene in liver tissue with fattening traits (* *p* ≤ 0.05; ** *p* ≤ 0.01).

Gene/Tissue.	*GPAT1*			*GPAT2*
Fattening Traits	m. longissimus lumborum	Subcutaneous Fat	Liver Tissue	Liver Tissue
Daily gain in performance test (g)	−0.070	−0.057	0.072	−0.310
Daily live gain (g)	−0.055	−0.231	−0.010	−0.302 *
Feed conversion ratio	0.032	0.178	−0.034	0.077
Daily feed intake (kg)	−0.046	0.066	0.044	−0.284
Age at slaughter (days)	−0.003	0.246 *	0.004	0.363 *
The number of days of control fattening (days)	0.010	0.066	−0.053	0.424 *

## Abbreviations:

The following abbreviations are used in this manuscript:

TAG	triacylglycerol
IMF	intramuscular fat
*GPAT1*	glicerol-3-phosphate acyltransferase 1
*GPAT2*	glicerol-3-phosphate acyltransferase 2
PL	polish landrace
PLW	polish large white
TPA	texture profile analysis
WBP	Warner–Bratzler shear force
WHC	water holding capacity
RFI	residual feed intake
